# One-Year Risk of Stroke After Transient Ischemic Attack or Minor Stroke in Hunter New England, Australia (INSIST Study)

**DOI:** 10.3389/fneur.2021.791193

**Published:** 2021-12-20

**Authors:** Shinya Tomari, Christopher R. Levi, Elizabeth Holliday, Daniel Lasserson, Jose M. Valderas, Helen M. Dewey, P. Alan Barber, Neil J. Spratt, Dominique A. Cadilhac, Valery L. Feigin, Peter M. Rothwell, Hossein Zareie, Carlos Garcia-Esperon, Andrew Davey, Nashwa Najib, Milton Sales, Parker Magin

**Affiliations:** ^1^Hunter Medical Research Institute, University of Newcastle, Newcastle, NSW, Australia; ^2^Department of Neurology, John Hunter Hospital, Newcastle, NSW, Australia; ^3^School of Medicine and Public Health, University of Newcastle, Newcastle, NSW, Australia; ^4^Warwick Medical School, University of Warwick, Warwick, United Kingdom; ^5^Health Service and Policy Research Group, University of Exeter, Exeter, United Kingdom; ^6^Faculty of Medicine, Nursing and Health Sciences, Monash University, Melbourne, VIC, Australia; ^7^Department of Medicine, University of Auckland, Auckland, New Zealand; ^8^Stroke and Ageing Research, School of Clinical Sciences at Monash Health, Monash University, Melbourne, VIC, Australia; ^9^Florey Institute of Neuroscience and Mental Health, University of Melbourne, Melbourne, VIC, Australia; ^10^National Institute for Stroke and Applied Neurosciences, Auckland University of Technology, Auckland, New Zealand; ^11^Nuffield Department of Clinical Neuroscience, Centre for Prevention of Stroke and Dementia, University of Oxford, Oxford, United Kingdom; ^12^Discipline of General Practice, University of Newcastle, Newcastle, NSW, Australia; ^13^Brunker Road General Practice, Newcastle, NSW, Australia

**Keywords:** transient ischemic attack, minor stroke, stroke-mimic syndrome, one-year risk of ischemic stroke, community-based study

## Abstract

**Background:** One-year risk of stroke in transient ischemic attack and minor stroke (TIAMS) managed in secondary care settings has been reported as 5–8%. However, evidence for the outcomes of TIAMS in community care settings is limited.

**Methods:** The INternational comparison of Systems of care and patient outcomes In minor Stroke and TIA (INSIST) study was a prospective inception cohort community-based study of patients of 16 general practices in the Hunter–Manning region (New South Wales, Australia). Possible-TIAMS patients were recruited from 2012 to 2016 and followed-up for 12 months post-index event. Adjudication as TIAMS or TIAMS-mimics was by an expert panel. We established 7-days, 90-days, and 1-year risk of stroke, TIA, myocardial infarction (MI), coronary or carotid revascularization procedure and death; and medications use at 24 h post-index event.

**Results:** Of 613 participants (mean age; 70 ± 12 years), 298 (49%) were adjudicated as TIAMS. TIAMS-group participants had ischemic strokes at 7-days, 90-days, and 1-year, at Kaplan-Meier (KM) rates of 1% (95% confidence interval; 0.3, 3.1), 2.1% (0.9, 4.6), and 3.2% (1.7, 6.1), respectively, compared to 0.3, 0.3, and 0.6% of TIAMS-mimic-group participants. At one year, TIAMS-group-participants had twenty-five TIA events (KM rate: 8.8%), two MI events (0.6%), four coronary revascularizations (1.5%), eleven carotid revascularizations (3.9%), and three deaths (1.1%), compared to 1.6, 0.6, 1.0, 0.3, and 0.6% of TIAMS-mimic-group participants. Of 167 TIAMS-group participants who commenced or received enhanced therapies, 95 (57%) were treated within 24 h post-index event. For TIAMS-group participants who commenced or received enhanced therapies, time from symptom onset to treatment was median 9.5 h [IQR 1.8–89.9].

**Conclusion:** One-year risk of stroke in TIAMS participants was lower than reported in previous studies. Early implementation of antiplatelet/anticoagulant therapies may have contributed to the low stroke recurrence.

## Introduction

Transient ischemic attack and minor stroke (TIAMS) account for up to 58% of all cerebral ischemic events (1, 2). Although recurrent vascular event rates post-TIAMS have been declining over the past two decades (3, 4), 5–8% of patients have disabling stroke, 1.1–1.3% have coronary artery events, and 0.6–0.7% die from cardiovascular causes in the year following TIAMS (5–7). Short-term dual antiplatelet therapy (DAPT) is the current gold standard for secondary prevention in high-risk TIAMS patients with non-cardioembolic sources (8). For those with atrial fibrillation, non-vitamin K antagonist oral anticoagulants (NOACs) are indicated as the first line therapy (9).

While the majority of TIAMS studies have been conducted in secondary-care settings such as hospital or specialist-care units, many TIAMS are managed in community-care settings. Short- and long-term outcomes of TIAMS in community-care settings have undergone more limited study, related to the fact that distinguishing TIAMS from TIAMS-mimics is challenging for non-neurologists (10).

## Aims

In the INternational comparison of Systems of care and patient outcomes In minor Stroke and Tia (INSIST) study (11), we aimed, (a) to establish rates of stroke, TIA, myocardial infarction (MI), and death post-index event for TIAMS and TIAMS-mimic groups recruited from primary-care population and, (b) to compare recurrence rates of stroke and TIA between both groups, and (c) to document antiplatelet and anticoagulant treatments in both groups pre-index event, at 24 h post-index event, and after 1 year.

## Methods

Ethic approval was gained from Hunter New England Human Research Ethics Committee (Reference No. 12/04/18/4.02). The participants provided written informed consent.

INSIST was a community-based prospective cohort study. The methods have been reported in detail in the protocol paper (11). TIA was defined as a neurological episode self-resolving in <24 h. Minor stroke was defined as a neurological deficit lasting >24 h with National Institute of Health Stroke Scale (NIHSS) ≤ 5 at presentation.

### Inclusion Criteria

- ≥18 years old.
- Suffered a possible TIAMS during the study period.
- Attended one of the participating practices.

### Exclusion Criteria

- Unable to provide informed consent e.g., cognitive impairment.
- Moderate/severe stroke at presentation (symptoms lasting more than 24 h and NIHSS >5).
- TIAMS but delayed consulting a general practitioner (GP) and subsequently presented with stroke or major vascular event.

### Study Population

We recruited consecutive patients between August 2012 and August 2016 from general practices in Hunter-Manning valleys regions of New South Wales, within the referral territory of acute neurovascular clinic at John Hunter Hospital (referral centre of Hunter New England Local Health District). Patients attended one of 16 general practices, of which 11 were in urban and five were in rural areas. Patients' eligibility was ascertained by multiple overlapping methods involving clinical records of general practices, after-hours GP service, the acute neurovascular clinic and emergency departments. The study population was possible TIAMS patients engaged with health systems at primary or secondary levels. While Australian evidence-based guidelines recommended optimal management of TIAMS to be urgent referral to acute specialist neurologist/stroke care (12), local practice was known to involve some TIAMS care being solely provided by GPs. Models of care included contributions from GPs, Emergency Departments, a dedicated Acute Neurovascular Clinic, inpatient admission, and specialist physicians or surgeons.

INSIST wasn't a community incidence study with ascertainment of all events (it didn't include events not presenting to medical care). Potential participants were recruited by invitation letters from the participating practices.

### Data Collection

Participants underwent a baseline interview, and follow-up interviews at three- and 12-months post-baseline assessment. Further data were collected from medical records at the study conclusion. Patients not consenting to participation (non-responders) had de-identified outcome data collected, including subsequent TIA, stroke, MI, coronary or carotid revascularization and death at 12 months.

### Outcome Measures

Outcomes were subsequent stroke, subsequent TIA, MI, coronary or carotid revascularization procedure, and death. Outcomes were assessed at 7-days, 90-days, and 1-year post-index event. Further outcomes were use of antiplatelet and anticoagulant medications pre-index event, at 24 h post-index event and after 1 year post-index event.

### Adjudication

An expert panel adjudicated the index events and subsequent events as stroke, TIA, or TIAMS-mimic using data from phone interviews and examination of GP clinical records including imaging findings. Determinations were blinded and cross-referenced with the Hunter Region Heart and Stroke Register (13). The panel consisted of three clinicians; experienced stroke physicians (CRL, HZ, CG-E) and GPs (PM, MS, AD), with at least one of each at meetings. TIAMS and TIAMS-mimic groups were defined based on the index event adjudication.

### Statistical Analysis

In descriptive analyses, continuous variables were summarized using mean with standard deviation or median with interquartile range (IQR). Clinical characteristics were compared between TIAMS and TIAMS-mimic participants using Pearson's chi-square test for categorical variables, Student's unpaired *t-*test for normally distributed continuous variables and Wilcoxon's rank sum test for non-normally distributed continuous variables.

Rates of the six events: subsequent stroke, TIA, MI, coronary or carotid revascularization and death within 7 days, 90 days, and 1 year, with 95% confidence intervals, were each estimated using the Kaplan-Meier (KM) estimator (6 events × 3 time points = 18 estimates). For subsequent stroke and TIA (considered separately), the time to event was compared between TIAMS and TIAMS-mimic group participants using log-rank tests. For participants who did not have a subsequent stroke or TIA within the defined study time, their survival time was right censored at final follow-up.

For each of the six events of interest, we also considered that the five alternative events may potentially alter the risk of the event of interest occurring and treated the alternative events as competing risks. For example, for the outcome of subsequent stroke, competing risks were defined as subsequent TIA, MI, carotid or coronary revascularization or death. For KM estimates and log-rank tests, this was achieved by censoring the survival time at the time of the competing risk for individuals experiencing a competing risk before the event of interest. Time to subsequent stroke and time to subsequent TIA were also compared between TIAMS and TIAMS-mimic group participants using competing risks regression (14) to estimate sub-distribution hazard ratios (SHR), defining competing risks as described above. The competing risks regression model was also used to plot the cumulative incidence of subsequent stroke and TIA.

Time from symptom onset to commencement or enhancement of antiplatelet or anticoagulant drugs was summarized using median with interquartile range. All statistical analysis was performed using STATA 15.0 (Stata Corp, Texas, USA) with significance level set at 0.05.

## Results

**Figure 1:**
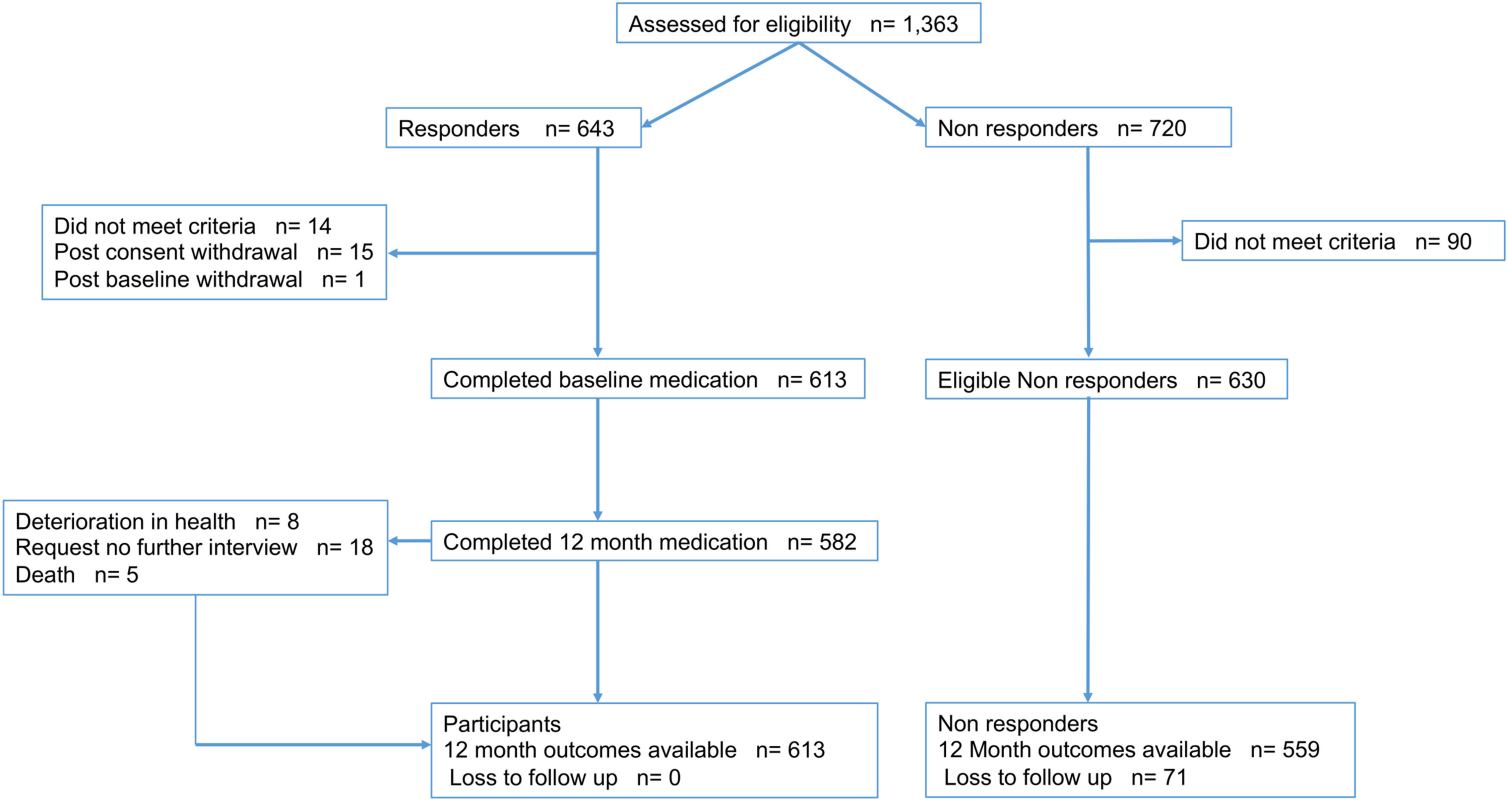
INSIST study enrolment.

Between August 2012 and August 2016, 1,363 patients were ascertained to have suffered a possible TIAMS event, with 643 consenting to participation (response rate 47%) and 613 meeting the study criteria (Figure 1). Outcome data were available in all participants, while medication use at 12 months was available in 582 (90.5%). Of 720 non-responders, 559 had clinical outcomes collected. Of 613 participants enrolled into the study, 278 (45%) were men, the mean age was 70 years, 298 (49%) were classified as TIAMS (175 TIA and 123 minor stroke −118 ischemic and 5 hemorrhagic) and 315 (51%) as TIAMS-mimics. Median time from symptom onset to seeing the first doctor was 19 [IQR 2.75–100] hours. Of TIAMS-group participants, 188 (63%) had an ABCD2 score ≥ 4, and 221 (74%) had secondary care/specialist management (26% managed solely by GPs). For TIAMS-mimic-group participants, 138 (44%) were managed solely by GPs (*P* < 0.001) (Table 1).

**Table 1:** Clinical characteristics of participating patients.

	**TIAMS**	**Mimics**	***p***
	***N* = 298**	***N* = 315**	
Age, years	72 ± 11	68 ± 13	<0.001
Gender, men	171 (57)	107 (34)	<0.001
Premorbid modified rankin scale 0–2	265 (89)	285 (90)	0.53
Secondary/specialist management	221 (74)	177 (56)	<0.001
**Medical history**			
Hypertension	223 (75)	202 (64)	0.004
Hyperlipidaemia	166 (56)	155 (49)	0.11
Diabetes/pre-diabetes	86 (29)	70 (22)	0.059
Atrial fibrillation	67 (22)	35 (11)	<0.001
Heart failure	33 (11)	18 (5.7)	0.016
Carotid/peripheral vascular disease	37 (12)	13 (4.1)	<0.001
Cardiovascular disease	93 (31)	61 (19)	0.001
Previous TIA/stroke	76 (26)	52 (17)	0.006
Current smoker	19 (13)	17 (12)	0.61
ABCD2 score^*^ ≥4	188 (64)	110 (35)	<0.001
Education <10 years	131 (44)	117 (37)	0.086
Living alone	78 (26)	70 (22)	0.25

*Data are presented as mean ± standard deviation or absolute (percentage) values*.

*TIAMS; transient ischemic attack and minor stroke*.

* *ABCD2 score; age, blood pressure, clinical features, duration of symptoms and history of diabetes*.

Descriptive analyses also showed TIAMS-group participants were more likely to be older (72 vs. 68 years, *p* < 0.001), have vascular risk factors—hypertension (75 vs. 64%, *p* = 0.004), atrial fibrillation (22 vs. 11%, *p* < 0.001), heart failure (11 vs. 5.7%, *p* = 0.016), carotid/peripheral vascular disease (12 vs. 4.1%, *p* < 0.001), cardiovascular disease (31 vs. 19%, *p* = 0.001) and previous TIA/stroke (26 vs. 17%, *p* = 0.006). The most common diagnosis of TIAMS-mimics was migraine (27%), followed by syncope (15%), and vestibular disease (12%).

### Clinical Outcomes

In the TIAMS group, there were three subsequent ischemic strokes (KM rate: 1%; 95% CI: 0.3, 3.1) and nine (3.0%; 1.6, 5.8) TIAs within 7 days post-index event, increasing to six (2.1%; 0.9, 4.6) and thirteen (4.4%; 2.6, 7.5) at 90 days, and to nine (3.2%; 1.7, 6.1) and twenty-five (8.8%; 6.0, 12.7) after 1 year (Table 2). Alternatively, the TIAMS-mimic group had one stroke and one TIA (0.3% each; 0.04, 2.2) within 7 days, increasing to two TIAs (0.6%; 0.2, 2.5) at 90 days, and two strokes (0.6%; 0.2, 2.5) and five TIAs (1.6%; 0.7, 3.8) after 1 year.

**Table 2:** Seven days, 90 days, and 1 year event outcomes after the index event.

	**7 days**				**90 days**				**One year**			
	**TIAMS**		**Mimics**		**TIAMS**		**Mimics**		**TIAMS**		**Mimics**	
	***n* (%)**	**[95% CI]**	***n* (%)**	**[95% CI]**	***n* (%)**	**[95% CI]**	***n* (%)**	**[95% CI]**	***n* (%)**	**[95% CI]**	***n* (%)**	**[95% CI]**
Ischemic stroke	3 (1.0)	[0.3, 3.1]	1 (0.3)	[0.04, 2.2]	6 (2.1)	[0.9, 4.6]	1 (0.3)	[0.04, 2.2]	9 (3.2)	[1.7, 6.1]	2 (0.6)	[0.2, 2.5]
TIA	9 (3.0)	[1.6, 5.8]	1 (0.3)	[0.04, 2.2]	13 (4.4)	[2.6, 7.5]	2 (0.6)	[0.2, 2.5]	25 (8.8)	[6.0, 12.7]	5 (1.6)	[0.7, 3.8]
Myocardial infarction	0	0	0	0	0	0	1 (0.3)	[0.05, 2.2]	2 (0.6)	[0.2, 3.1]	2 (0.6)	[0.2, 2.6]
Coronary revascularization	0	0	0	0	2 (0.7)	[0.2, 2.9]	2 (0.6)	[0.2, 2.5]	4 (1.5)	[0.6, 4.0]	3 (1.0)	[0.3, 3.0]
Carotid revascularization	1 (0.3)	[0.05, 2.4]	0	0	10 (3.5)	[1.9, 6.4]	0	0	11 (3.9)	[2.2, 6.9]	1 (0.3)	[0.05, 2.2]
Death	0	0	0	0	0	0	0	0	3 (1.1)	[0.4, 3.5]	2 (0.6)	[0.2, 2.6]

*Data are presented as absolute values, (rates) and [95% CI]*.

*CI, confidence interval; TIAMS, transient ischemic attack and minor stroke*.

**Figure 2:**
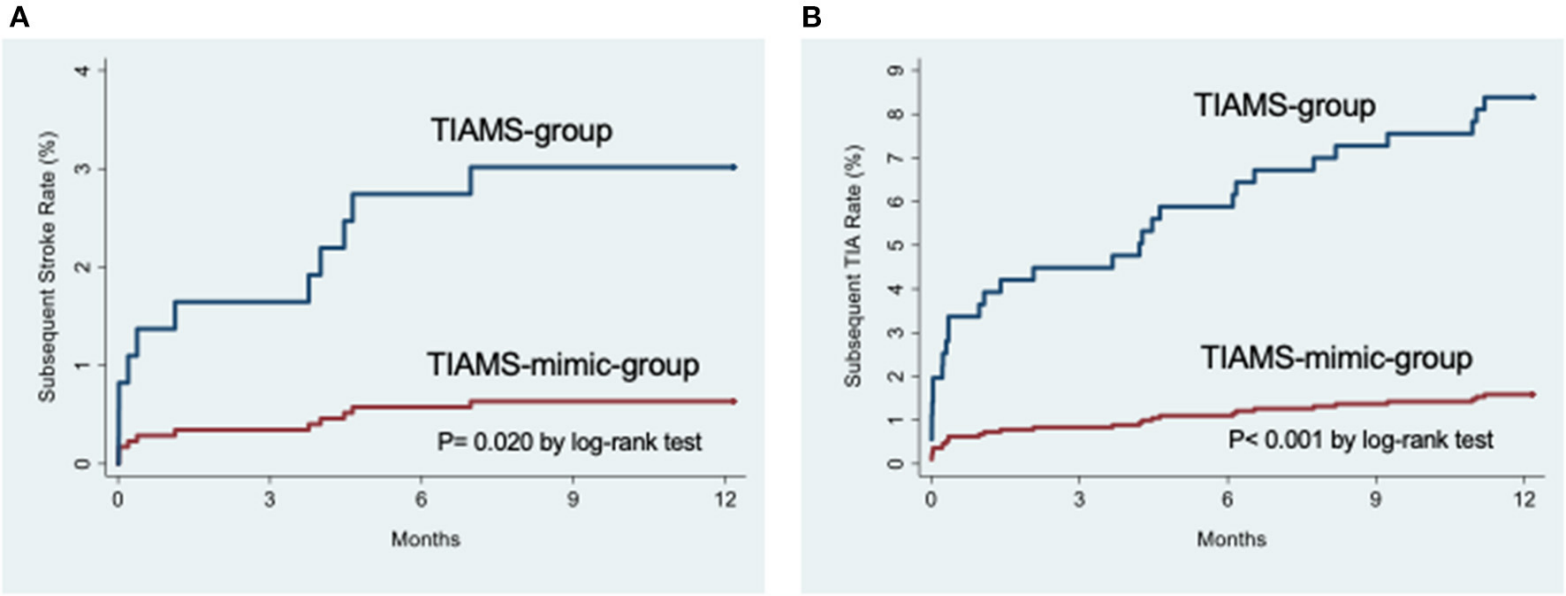
**(A)** Cumulative incidence functions for subsequent stroke from the time of the index event. **(B)** Cumulative incidence functions for subsequent transient ischemic attack from the time of the index event.

Rates of subsequent stroke and TIA were higher among TIAMS-group than TIAMS-mimic-group participants (*p* = 0.020 for stroke and *p* < 0.001 for TIA, from log-rank tests) (Figure 2). The estimated SHR with 95% CI for subsequent stroke in TIAMS-group vs. TIAMS-mimic-group participants, accounting for competing risks, was 4.8 (1.04, 22.3; *p* = 0.045) and for subsequent TIA was 5.5 (2.1, 14.3; *p* = 0.001); CIs were wide due to low event rates. The proportional hazards assumption was met for both models. There were two MI events in each group (KM rate: 0.6%) after 1 year. Four TIAMS-group and three TIAMS-mimic-group participants had coronary revascularization, and eleven TIAMS-group and one TIAMS-mimic-group participants underwent carotid revascularization. Three TIAMS-group and two TIAMS-mimic-group participants died from non-cardiovascular causes; Table 2 shows KM rates for all events. No intracerebral or subarachnoid hemorrhage were recorded. Non-responders had five strokes (0.9%), seventeen TIAs (3.0%), seven MI events (1.3%), three coronary revascularization (0.5%), two carotid revascularization (0.3%), and seventeen deaths (3.0%) during the 12 months follow-up.

### Secondary Prevention

**Figure 3:**
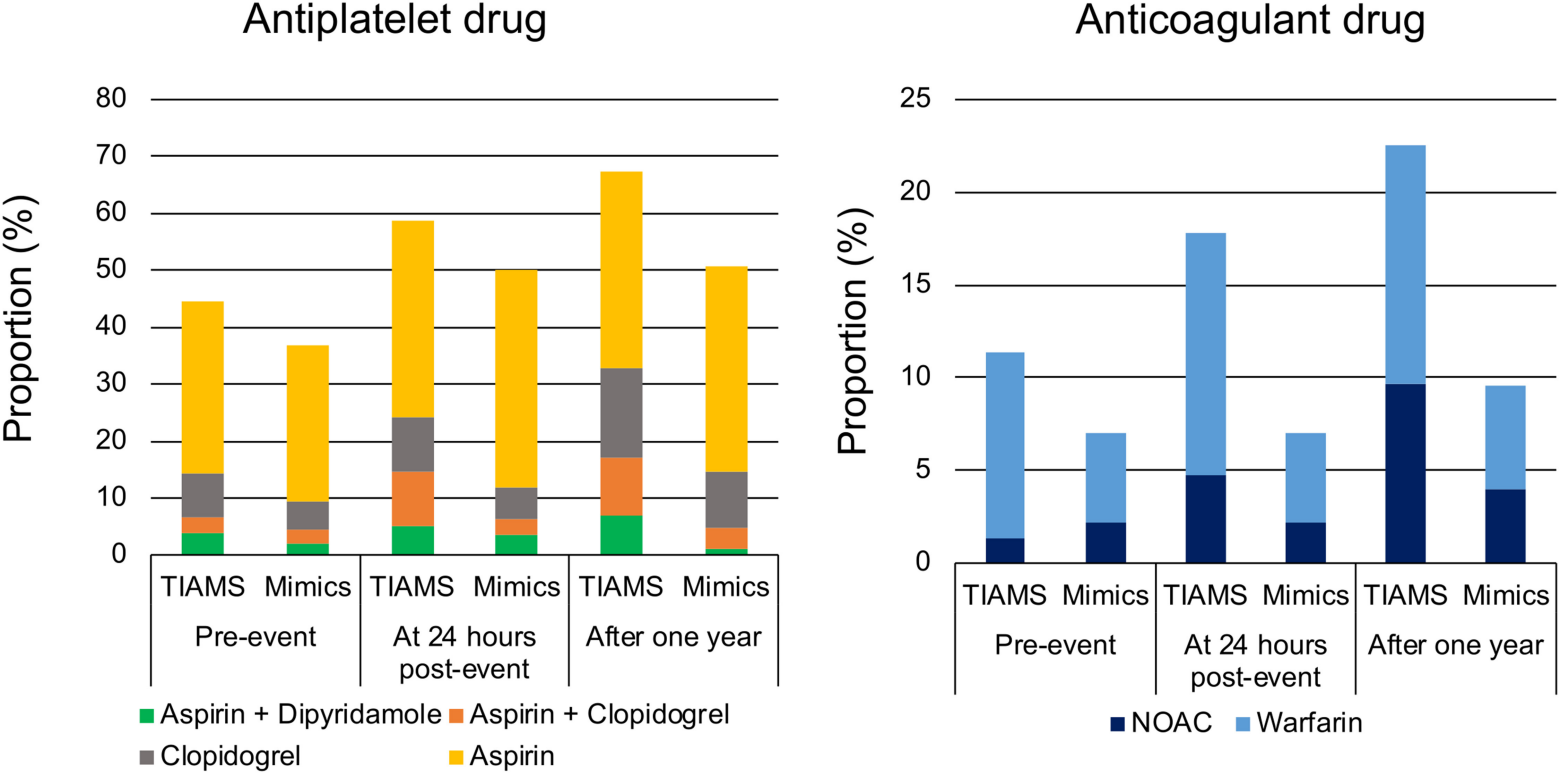
Antiplatelet and anticoagulant drugs pre-index event, at 24 h post-index event and after 1 year in TIAMS- and TIAMS-mimic-group participants (these are simple proportions). TIAMS, transient ischemic attack and minor stroke; NOAC, non-vitamin K antagonist oral anticoagulant.

Of 298 TIAMS-group participants, 133 (45%) were using antiplatelet therapy pre-index event, increasing to 178 (60%) at 24 h post-index event with 103 (35%) treated with aspirin alone (Figure 3). Of 315 TIAMS-mimic-group participants, 116 participants (37%) were using antiplatelet therapy pre-index event, increasing to 158 (50%) at 24 h with 121 (38%) treated with aspirin alone. There was a marked increase in DAPT in TIAMS group, increasing from 20 participants (6.7%) pre-index event to 44 (15%) at 24 h, compared to 14 participants (4.4%) increasing to 20 (6.7%) in TIAMS-mimic group. Anticoagulant therapy was used in 34 (11%) TIAMS-group participants pre-index event, increasing to 53 (18%) at 24 h post-index event, but was unchanged in TIAMS-mimic group with 22 (7%). NOACs use increased from four (1.3%) TIAMS-group participants pre-index event to 14 (4.7%) at 24 h, but was unchanged in TIAMS-mimic group with seven (2.2%). Of all participants, 275 (167 TIAMS and 108 mimics) commenced or received enhanced antiplatelet/anticoagulant therapy post-index event.

Of TIAMS-group participants who commenced or received enhanced therapies post-index event, 95 (57%) were treated within 24 h, compared to 48 (44%) of TIAMS-mimic group. Among the 275 participants managed with new or enhanced therapies, time from symptom onset to the therapies in TIAMS group was shorter than in TIAMS-mimic group (median 9.5 [IQR 1.8–89.9] hours vs. 31.7 [4.9–133.4] hours, *p* < 0.001).

## Discussion

We report a subsequent stroke rate in TIAMS participants at 7-days of 1% (95% CI: 0.3, 3.1), which can be compared to earlier studies of 2.1–3.4% (5, 15), a 90-days stroke risk of 2.1% (CI: 0.9, 4.6) compared to 1.2–7.4% (3–5, 15–17), and a 1-year rate of 3.2% (CI: 1.7, 6.1) compared to 5.1–8.1% (4, 5, 7). A subsequent TIA rate in TIAMS participants at 1-year of 8.8% was similar to previously reported 7.4% (5).

INSIST was an observational study of unselected patients of general practices including patients exclusively managed in general practices—but the proportion of participants with stroke recurrence appeared comparable or lower with those of study populations managed in secondary/specialist clinics [2.1% at 7-day (5), 1.2% (16), 2.1% (17), and 3.7% (5) at 90-day, and 5.1% at 1 year (5)].

We can hypothesize several reasons for the lower recurrence rate, but the most compelling is the prompt institution of antiplatelet/anticoagulant therapy post-index event. This was particularly so for aspirin in early post-TIAMS period (18). Time from symptom-onset to the therapies (median 9.5 hours [IQR 1.8–89.9]) was shorter than previously reported; time from seeking medical-attention to first-prescription (median 1 day [IQR 0–3]) (19). High community awareness in our study region may have led to the rapid treatment for TIAMS patients. National Prescribing Service MedicineWise Stroke Prevention Program implemented across Australia in 2009–2010 (20) might be correlated with high TIAMS awareness in GPs.

Another consideration is that our study population may have included TIAMS with better prognoses than previous studies. However, more than 60% of our TIAMS participants had an ABCD2 score ≥ 4, which was similar in the TIAregistry.org project (5) where systems were dedicated to urgent specialist evaluation. In the Framingham study with comprehensive community ascertainment of incident TIAs (4), 5.9% of participants at 90-days and 7.6% at 1-year had strokes. This suggests that TIAs with good prognoses being more likely to be managed in primary than secondary care, is unlikely to be a factor in our findings. The low incidence of stroke in non-responders suggests that our study did not lack TIAMS patients with poor prognoses at the enrolment.

Our findings provide further insight into GPs' approach to possible TIAMS. Aspirin in the TIAMS-mimic group at 24-h post-event was comparable to the TIAMS group. There was no corresponding increase in DAPT or anticoagulant use. GPs recognize that TIAMS is a medical emergency and differentiation of TIAMS and TIAMS-mimics is difficult. Therefore, if GPs can't exclude TIAMS, they may elect a “minimalist” approach of aspirin-only management rather than DAPT (though noting that for much of our study period DAPT was not yet guideline-recommended). Aspirin use for TIAMS/TIAMS-mimics may represent a liberal instance of recommended urgent prescription at diagnosis for suspected TIA (21).

### Study Strengths and Limitations

INSIST was a comprehensive community-based study, reflecting real-world TIAMS management by clinicians including exclusive management by GPs. Overlapping means of TIAMS/TIAMS-mimics ascertainment was rigorous. With 16 participating GP practices, including urban and rural settings, the results are broadly generalizable to the Australian general practice setting and to other health systems where GPs are gatekeepers to secondary care. There are several limitations. The response rate of 47% was modest, but it is a reasonable response rate for cohort studies (22). And we collected de-identified outcome data for non-responders. Medication data after 1 year was missing in 31 participants, however outcome data was complete. Reported HRs represented univariable estimates only because the event count was low, and we did not include any adjustment variables in the Cox model.

### Implications for Practice

Australian guidelines recommend urgent specialist-care for suspected TIAMS at high risk (12). TIAMS outcomes in our study were favorable even with 26% of TIAMS participants managed solely by GPs. 35% of TIAMS participants treated with aspirin alone at 24 h post-index event may reflect the then-current guidelines without evidence for DAPT. The majority of TIAMS-mimic participants treated with aspirin alone may reflect diagnostic uncertainty among GPs. Lack of full confidence in the diagnosis may deter introduction of DAPT and transfer to specialist/secondary care. Therefore, GP access to specialists may be desirable for diagnostic or management advice. Given multiple barriers to access to specialist-care (23), an appropriate response may be institution of TIAMS “rapid response” telehealth service (24, 25).

## Conclusion

We have established the recurrent stroke and major vascular event rates after TIAMS in a regional community-based Australian healthcare setting. The recurrence rate appears to be comparable with other TIAMS cohorts from specialist/secondary care settings. Rapid prescription of new or additional antiplatelet/anticoagulant drugs may have contributed to the low recurrence rate. However, our data suggest scope for improvements in the immediate management of TIAMS in the Australian regional healthcare setting.

## Data Availability Statement

The raw data supporting the conclusions of this article will be made available by the authors, without undue reservation.

## Ethics Statement

The studies involving human participants were reviewed and approved by Hunter New England Human Research Ethics Committee. The patients/participants provided their written informed consent to participate in this study.

## Author Contributions

CL, DL, JV, PAB, DC, and PM: study design. HZ, CG-E, AD, NN, and MS: data collection. EH: statistical analysis. ST, CL, and PM: interpreted the data. ST, CL, JV, HD, PAB, NS, VF, PR, and PM: drafted and revised the manuscript. All authors contributed to the article and approved the submitted version.

## Funding

The INSIST study was funded by NHMRC Project Grant ID 1027794.

## Conflict of Interest

NS was the recipient of co-funded National Health and Medical Research Council Career Development/National Heart Foundation Future Leader Fellowship (APP1110629/100827). VF's work was partly funded by the Health Research Council of New Zealand. The remaining authors declare that the research was conducted in the absence of any commercial or financial relationships that could be construed as a potential conflict of interest. The handling editor declared a shared affiliation with author PR at time of review.

## Publisher's Note

All claims expressed in this article are solely those of the authors and do not necessarily represent those of their affiliated organizations, or those of the publisher, the editors and the reviewers. Any product that may be evaluated in this article, or claim that may be made by its manufacturer, is not guaranteed or endorsed by the publisher.
